# Automatic Detection of Image-Based Features for Immunosuppressive Therapy Response Prediction in Oral Lichen Planus

**DOI:** 10.3389/fimmu.2022.942945

**Published:** 2022-06-23

**Authors:** Ziang Xu, Qi Han, Dan Yang, Yijun Li, Qianhui Shang, Jiaxin Liu, Weiqi Li, Hao Xu, Qianming Chen

**Affiliations:** ^1^ State Key Laboratory of Oral Diseases, National Clinical Research Center for Oral Diseases, Chinese Academy of Medical Sciences Research Unit of Oral Carcinogenesis and Management, West China Hospital of Stomatology, Sichuan University, Chengdu, China; ^2^ Key Laboratory of Oral Biomedical Research of Zhejiang Province, Affiliated Stomatology Hospital, Zhejiang University School of Stomatology, Hangzhou, China

**Keywords:** oral lichen planus, immunosuppressive therapy, image-based feature, prediction, oral mucosa

## Abstract

Oral lichen planus (OLP) is a chronic inflammatory disease, and the common management focuses on controlling inflammation with immunosuppressive therapy. While the response to the immunosuppressive therapy is heterogeneous, exploring the mechanism and prediction of the response gain greater importance. Here, we developed a workflow for prediction of immunosuppressive therapy response prediction in OLP, which could automatically acquire image-based features. First, 38 features were acquired from 208 OLP pathological images, and 6 features were subsequently obtained which had a significant impact on the effect of OLP immunosuppressive therapy. By observing microscopic structure and integrated with the corresponding transcriptome, the biological implications of the 6 features were uncovered. Though the pathway enrichment analysis, three image-based features which advantageous to therapy indicated the different lymphocytes infiltration, and the other three image-based features which bad for therapy respectively indicated the nicotinamide adenine dinucleotide (NADH) metabolic pathway, response to potassium ion pathway and adenosine monophosphate (AMP) activated protein kinase pathway. In addition, prediction models for the response to immunosuppressive therapy, were constructed with above image-based features. The best performance prediction model built by logistic regression showed an accuracy of 90% and the area under the receiver operating characteristic curve (AUROC) reached 0.947. This study provided a novel approach to automatically obtain biological meaningful image-based features from unannotated pathological images, which could indicate the immunosuppressive therapy in OLP. Besides, the novel and accurate prediction model may be useful for the OLP clinical management.

## Introduction

Oral lichen planus (OLP) is a common chronic inflammatory disease of the oral mucosa with a prevalence of 0.5% to 2.0% in the general population (1). OLP mainly affects the middle-aged population (50-60 years old), more commonly women (2). OLP can manifest clinically as reticular, popular, plaquelike, erosive, atrophic, and bullous subtypes (3). Oral lesions in the form of atrophic erosions of OLP can cause symptoms ranging from a burning sensation to severe pain that interferes with speech, eating and swallowing (4). It has been known that T cell-mediated cytotoxicity is involved in pathogenesis of OLP (5). Therefore, immunosuppression is commonly used to treat OLP (6, 7). However, not all patients show positive response to immunosuppressive therapy. The commonly used therapeutic agents are corticosteroids, calcineurin inhibitors, which mainly work by inhibiting immunity and inhibiting lymphocytes (8). Pathological images can reveal many immune-related changes. With the help of pathological images, we can discover the factors that affect the treatment effect.

With the recent advent of cost-effective digital scanners for full slides, tissue slides can be digitized and stored as digital images (9). Digital pathology has made the computerized quantitative analysis of histopathological images possible. Deep learning (DL) is increasingly being used in medical images, including diagnosing and grading tumors (10–12), prognosis and prediction of metastasis (13, 14). However, deep learning requires a large amount of annotated data for training (15), including pixel-level annotated datasets and labeled datasets. These deep learning practices have met with varying degrees of success, and Google has also announced the development of microscopes based on deep learning algorithms to assist pathologists in diagnosis (16). These deep learning practices have better performance in the field of pathological images and can achieve an accuracy up to 97.51% in the classification of tumor differentiation grade (17).

However, due to the uninterpretability of DL algorithms, the opacity of artificial intelligence (AI) decisions is one of the most significant challenges to their regulatory approval and clinical implementation (18). Despite of the high accuracy rates that can be achieved by algorithms, their credibility is still questionable for practical clinical applications. Especially in oncology issues, the adoption of unexplained AI systems may raise severe legal and ethical challenges, including regulatory difficulties and confusion over the allocation of responsibilities (19). In addition, although deep learning algorithms can be trained to explore features that are difficult for pathologists to find, we do not know exactly what these features are. Therefore, it is risky to make clinical decision just rely on deep learning algorithms (20).

Autoencoder is an unsupervised feature extraction method (21). This method can be used for dimensionality reduction representation of high-dimensional images (22, 23). Generally, autoencoders can be considered as neural network models with a multilayer structure, consisting of two parts: encoding and decoding. The encoder encodes the input image data, while the decoder decodes the expressions in the hidden layer to reconstruct the input data. Thus, the data in the hidden layer contains the core information of an image (24). By clustering the hidden layer data of a large number of images can discover potential correlations between images (25). We can group images with similar features into one class by compressing and clustering the image information. If most of the images within a feature are from patients who achieve great clinical efficacy with immunosuppressive treatment, it means that the feature may be relevant to immunosuppressive treatment.

Based on the above issues, we developed a method to obtain interpretable knowledge from unlabeled pathological images and applied our method to obtain features that have positive/negative effects on the immunosuppressive treatment of OLP. Further, we determined the significance of these features by analyzing the biological phenotype and RNA-seq of these features. Finally, we constructed a model to predict the efficacy of immunosuppressive treatment for OLP. The pathological features identified in our study may be informative for clinical treatment.

## Materials and Methods

### Study Participants

This hospital-based cohort consisted of OLP patients who attended the mucosal unit at West China Dental Hospital, Sichuan University from September 2019 to September 2021. Participants all signed written consent to participate in the study. The study was approved by the Ethics Committee of West China Dental Hospital of Sichuan University (WCHSIRB-D-2017-021).

Patients attending the study were screened for inclusion criteria. The detailed criteria are shown in **Table 1**.

**Table 1:** The include exclusion criteria.

Inclusion criteria
Signed informed consent.
Age 18-65 years old, Han nationality.
**Clinically:** 1. There are bilateral lesions with a certain degree of symmetry. 2. There is a lace-like reticulation (reticular type) composed of gray-white lines slightly above the mucosal surface. 3. It can be manifested as erosive, atrophic, bullous and plaque-type lesions, but there must be a reticular type in other parts of the oral mucosa. **Pathologically:** 1. Confined to the surface of the connective tissue, a well-defined band-like cell infiltration area, mainly lymphocytes. 2. Basal cell liquefaction degeneration. 3. No epithelial dysplasia.
**Exclusion criteria**
Pregnant women, lactating women, women of childbearing age who plan to become pregnant within 1 year.
Patients with obvious other oral mucosal diseases or severe periodontitis (periodontal pocket>6mm, attachment loss>5mm, alveolar bone resorption more than 1/2 of the root length)
Patients with major infectious diseases (such as AIDS, syphilis, etc.), or other precancerous lesions or tumors.
Patients with a history of immune system disorders (e.g., systemic lupus erythematosus, rheumatoid arthritis, scleroderma, hyperthyroidism, ulcerative colitis, etc.).
Patients who have received immunotherapy within 3 months.
Abnormal liver function: (ALT elevation greater than 1.5 times the upper limit of normal); renal dysfunction (creatinine and/or blood urea nitrogen or urea elevation greater than 1.5 times the upper limit of normal); hemolytic anemia, thrombocytopenia (PLT < 60 ×10^9^/L), white blood cells <3 × 10^9^/L, neutrophils <1.5 × 10^9^/L; central or peripheral nervous system involvement.
Patients with abnormal ophthalmic examinations (including visual acuity, slit lamp examination, ophthalmoscopy, and visual field examination).
Glaucoma and cataract patients.
Those who are allergic to the ingredients of hydroxychloroquine sulfate tablets.
The lesioned mucosa corresponds to those filled with amalgam in the tooth.
Other factors for which the investigator considered subjects unsuitable for participation in this study.

The total number of participants who met the criteria was 56. Immunosuppressive treatments were applied for these participants, including oral hydroxychloroquine sulfate tablets and dexamethasone sodium phosphate injection. We collected complete pathological sections and clinical data from all patients for analysis. The scores were according to the presence of three types of white reticulations/patches, erythema/congestion, and erosions/ulcers. In the 11 parts of the mouth: upper lip, lower lip, left cheek, right cheek, maxillary gum, mandibular gum, left tongue, right tongue, floor of the mouth, hard palate, and soft palate, the score was 0 if it was not present, 1 if it was present but not more than 50% of the area of the part, and 2 if it was more than 50%. The total score was finally calculated. The calculation was weighted 1.5 times for the erythema/congestion score and 2 times for the erosion/ulcer score. We followed up the patient three times, and if the score at the third time was more than 10% lower than the first time, the patient was considered effective for immunosuppressive treatment. Among them, 19 patients were ineffective with immunosuppressive treatment and 37 were effective.

### Preparation of Whole-Mount Pathology Images and RNA Sequencing

The tissue sample from patients would be divided into two halves, one for pathology section preparation and the other for RNA-seq. Understanding the gene expression of the corresponding pathological image is essential to interpreting the functional components of the genome, and revealing the molecular composition of cells and tissues, as well as understanding development and disease. RNA-seq can be used to study the transcriptome (26). The transcriptome is the complete set of transcripts and their number in cells at specific developmental stages or physiological conditions. Understanding the transcriptome is essential for interpreting the functional components of the genome and revealing the molecular composition of cells and tissues, as well as for understanding development and disease. In this study, tissues from 19 patients were sent for RNA-seq to interpret the biological implications of the image-based features.

### Preprocessing of Data Set

We divided the 168 whole slide images (WSIs) from 56 patients into the following two groups: 38 WSIs from 38 patients (the images with the largest number of pixels selected for each patient) were used to generate key features using the deep neural network, and the other 130 WSIs were used to validate the prediction model by using these features. We did not provide any information about the patients to the deep neural network during the training process. In addition, the pathologists did not examine or annotate the pathology images. For training and feature extraction of 38 patients, the number of patients with good and poor results for immunosuppressive treatment was the same (n=19).

In this study, we used Python’s Openslide (version 1.1.2) to perform the segmentation process on WSIs. The parameters we choose are a magnification of 50x and a patch size of 128*128. We cut the WSIs without gaps into patches of 128*128 pixels size. 38 WSIs from 38 patients were segmented into the original patches set. During the production of pathology sections and electronic scans, some images showed contamination and distortion, so we performed an additional filtering step on the original patches set. Based on the characteristics of our dataset, our filtering criteria included: a. Blank area ≥ 75% (too little valid content); b. Average brightness of all pixels in the image < 120 or > 250 (image too dark/too bright); c. Variance of RGB values of all pixels in the image < 80 (image with contamination); d. Width or height less than 128 pixels (image at the edge of the slice). After the above screening, patches that are not suitable for training and feature extraction are removed, while the normal patches were reserved.

### Autoencoder

**Figure 1:**
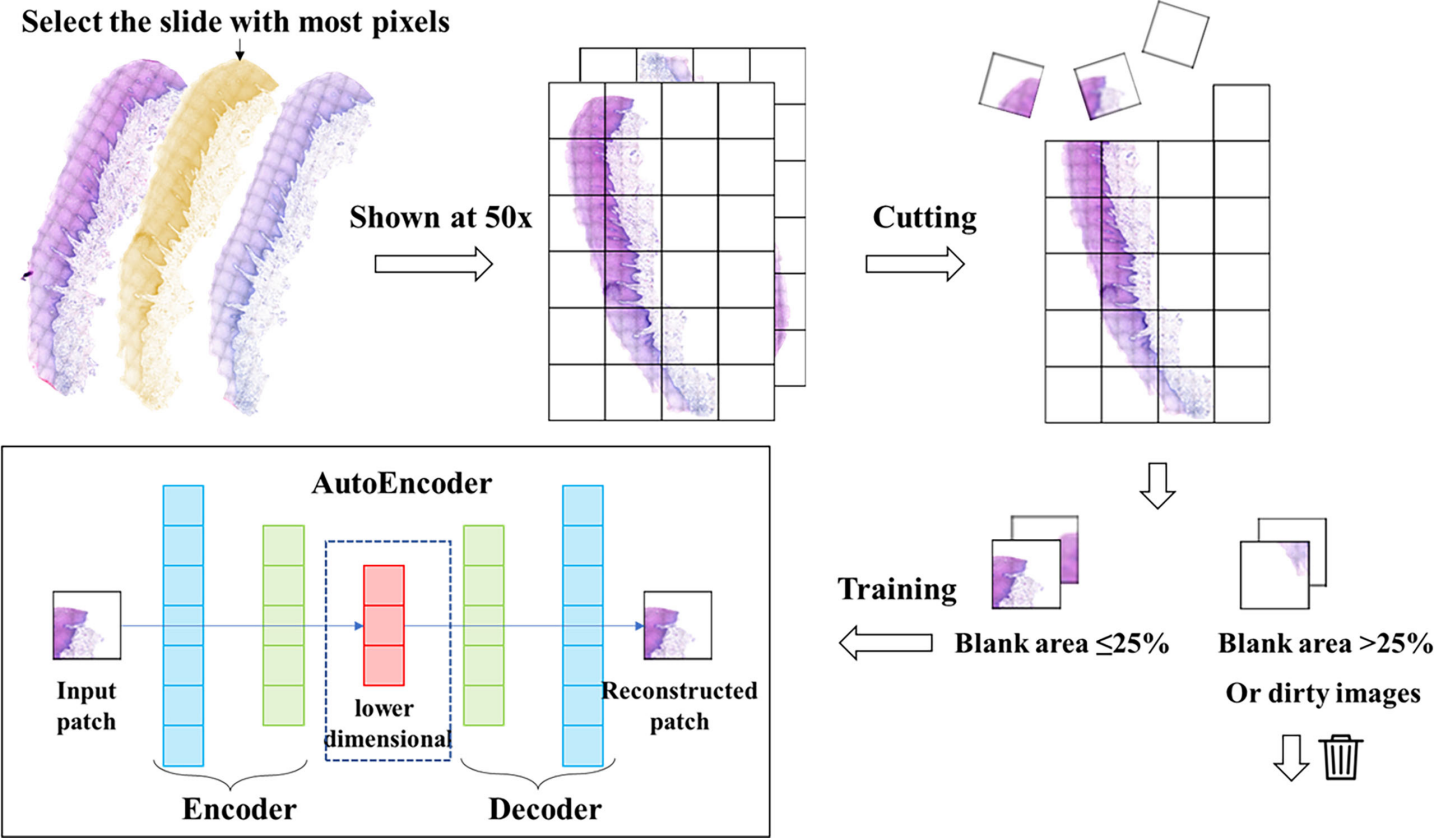
Data processing and autoencoder construction. We selected the slide with the largest number of pixels from each patient’s slide and cut it into patches of 128*128 pixels. Then we filtered these patches to remove the patches with low quality, and then trained the autoencoder with the filtered patches set. The trained autoencoder can compress the image into a one-dimensional vector.

Autoencoder consists of two main parts: encoder and decoder. The encoder compresses the input data to obtain low-dimensional data containing key information of the original data (22). The decoder can decode these obtained data and restore them to the original data through the decoding process. The training process consists of three main steps. In the first step, the encoder encodes the unlabeled data samples and obtains the encoding code. In the second step, the decoder decodes the encoded codes and obtains the new data. In the third step, we calculate the information error between these new data and the original data, and then adjust the weight parameters of the encoder and decoder according to the error to minimize the reconstruction error (27). For our encoder, it is possible to downscale an image with 128*128 pixels and three-channel values (RGB values) into a vector of 2048 numbers. The method we used does not require manual annotation or labeling to pre-classify the image. The entire training process is unsupervised, and Step1 of **Figure 1** provides a flowchart for training the autoencoder network.

### Image-Based Feature Generation Method

We used a set of filtered patches (n=122,705) to train the autoencoder. A total of 50 epochs were trained. and the vectors in the middle layer were clustered using K-means clustering methods. Clustering includes several machine learning algorithms that attempt to identify similar data instances and group them together (28). Algorithms such as K-means try to group the data around a point (called the center of mass), while other algorithms try to find clusters hierarchically. In total, 38 features (clusters) were generated.

At this point, each patch is assigned a label, and if the treatment effect of the WSI corresponding to the patient is good, then the patch is defined as positive. Otherwise, it is defined as negative. Next, we find the centroids of each feature in the k-means generation process. The scores *u_i, j, k_* are calculated based on the distances *d_i, j, k_* (the distance from the *j*th patch of the *i*th patient to the *k*th feature) between this vector and each centroid, which we define using the following simple method (25):


(1) $$u_{i,\;j,\;k}\;=\;1\;\;if\;argmin\;d_{i,\;j,\;k}\;and\;0\;otherwise\;\left(k=1,\;2,\;3,\;\ldots ,38\right)$$

The positive and negative degrees of each feature are defined as follows, +*u_i, j, k_* and -*u_i, j, k_* refers to the number of positive and negative patches in the feature, *n_+_* and *n_-_* The number of positive and negative patches among all the patches involved in clustering.


(2) $$r_{+,\;k}\;=\;\frac{\Sigma +u_{i,\;j,\;k}}{n_{+}}\;\;\left(k=1,\;2,\;3,\;\ldots ,38\right)$$

(3) $$r_{-,\;k}\;=\;\;\frac{\Sigma -u_{i,\;j,\;k}}{n_{-}}\;\;\;\left(k=1,\;2,\;3,\;\ldots ,38\right)$$

Finally, we define the impact score *I_k_* of the kth feature as:


(4) $$I_{k}\;=\;\frac{r_{+,\;k}}{r_{+,\;k}+r_{-,\;k}}$$

The value of *I_k_* ranges from 0 to 1, and the contribution of different features to the prediction results varies when constructing the prediction model. We define the weight of the *k*th feature as


(5) $$W_{k}=\;1+\;\left|\;0.5-\;I_{k}\;\right|$$

At the end of clustering, we identify the biological phenotypes of all features and filter out the features that contribute significantly to the treatment effect, called key features.

### Construction of Predictive Models

The clustering is performed by K-means to obtain 38 clustering results, each with a centroid *K_1_*, …, *K_38_*. After the input of the slide to be predicted, we cut and filter it (in “key feature generation method”) to obtain the patches set. The trained encoder encodes the patches into a vector *X_i_* and calculates the Euclidean distance from the vector to the centroid of each cluster. The cluster with the smallest distance is the class of the patches.

We mainly used two methods, logistic regression and SVM, to build prediction models to evaluate the value of using 38 features to predict the treatment effect. SVM is a powerful method to build classifiers. It aims to create a decision boundary between two categories so that labels can be predicted from one or more feature vectors (29). This decision boundary is called the hyperplane, and it is oriented as far as possible from the nearest data points in each class. These closest points are called support vectors.

To evaluate our method, we used 38 features generated by deep learning to predict cancer recurrence. To address the fact that feature values are unevenly distributed between patients with and without cancer recurrence, we multiplied each feature value by *W_k_* (see the Methods section for key feature generation methods), which enhanced the predictive power of the model (25). Due to the small sample size used for validation, we adopted 10-fold cross validation. A data set is first randomly divided into 10 disjoint folds that have approximately the same number of instances. Then every fold in turn plays the role for testing the model induced from the other 9 folds. Since the partition is random, the variance of the accuracy estimates can be large for statistical inference (30). We use Receiver Operating Characteristic (ROC) and accuracy to compare models generated by deep learning (31). The area under the receiver operating characteristic (AUROC) curves is the most commonly used metric for comparing classifier performance and takes values from 0 to 1. The higher the AUC, the better the performance of model (32).

### Pathway Enrichment Analysis

Using the median number of generated key feature k present in all pathology sections of the training set as the session, all pathology sections of the training set were divided into feature k high expression and low expression groups. We performed gene set variation analysis (GSVA) using raw counts to identify signaling pathways and functions that were significantly enriched using in each group. The input for the GSVA algorithm is a gene expression matrix in the form of RNA-seq counts and a database of gene sets. The output of the algorithm is a matrix containing pathway enrichment scores for each gene set and sample (33).

### Statistical Analysis

We used Python (version 3.8) for data processing and model training, OpenSlide package (version 1.1.2) and OpenCV-Python package (version 4.3.0.36) for processing and cutting of WSIs, and TensorFlow (version 2.3.0), NumPy (version 1.18.4) and PIL (version 8.2.0) for training of deep autoencoders and processing of intermediate layers. NumPy (version 1.18.4) and PIL (version 8.2.0). The clustering and pathway analysis of the intermediate layer data were performed by using R, including e1071 (version 1.7.0), GSVA (version 1.20.0). All packages are available. All tests were two-tailed and considered statistically significant if the p-value < 0.05.

## Result

### Deep Autoencoder

We developed a method for automatic feature generation based on autoencoders. This method used an unsupervised neural network. No direct information about the cancer was provided to the deep neural network. The encoder can downscale an image of 128*128 pixels into a vector containing 2048 numbers. The workflow of data processing and auto-encoder was shown in **Figure 1**. The decoder can recover the vector into an image. The recovered image is basically the same as the original image. We used the encoder to encode 122,705 patches of the filtered patches set into a vector set

### Clustering Workflow and Features Analysis

**Figure 2:**
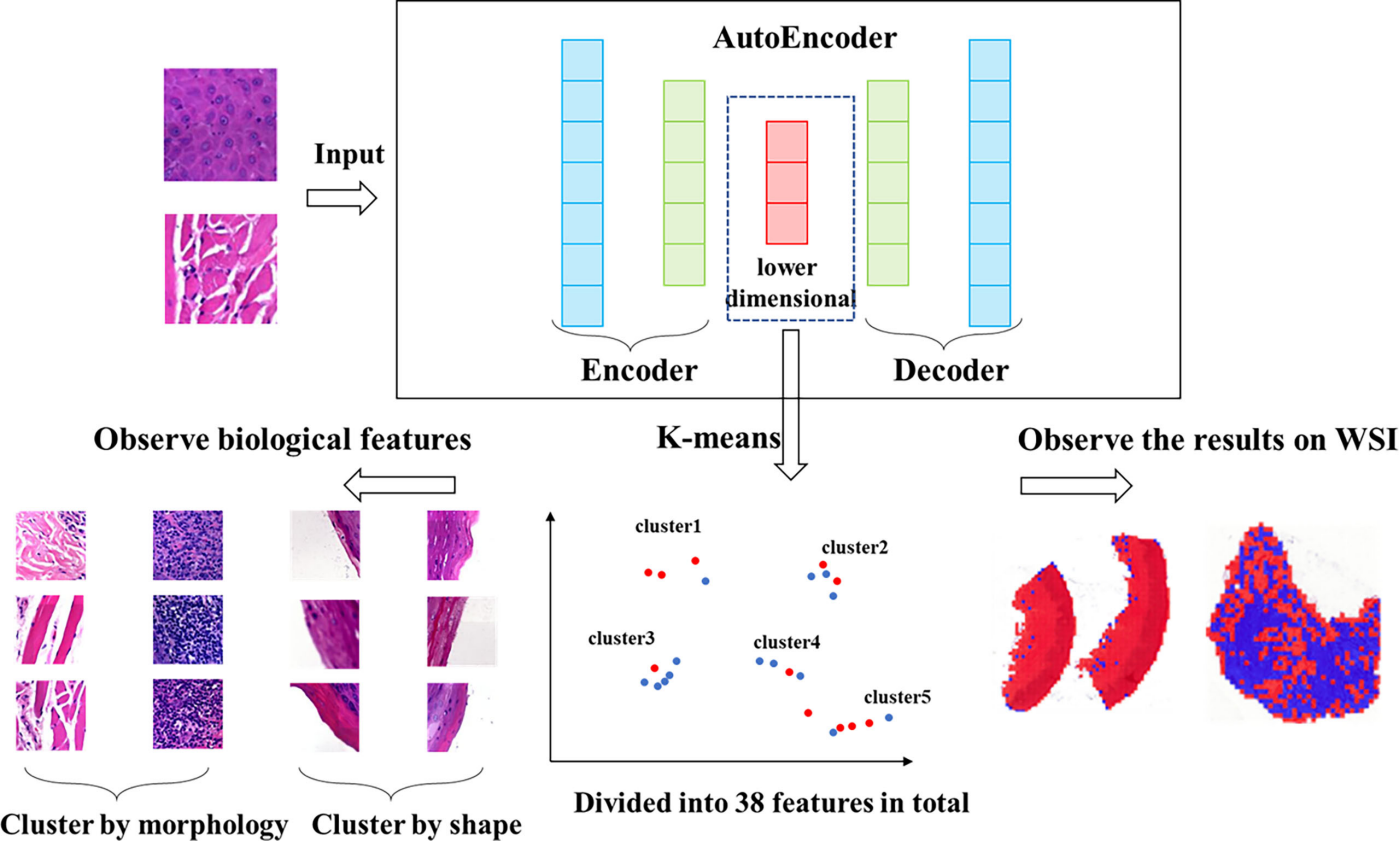
Workflow of clustering. We clustered the data, which was encoded by autoencoder, obtained 38 features *via* K-means method. The higher the proportion of positive patches contained in feature, the greater the positive significance of the effect of immunosuppressive treatment. The higher the proportion of negative patches, the greater the negative significance of the treatment effect.

The vector set was clustered by K-means method. A total of 38 clusters were generated, which we called features. We calculated the *I_k_* value of each feature and evaluated the role of each feature in the effect of immunosuppressive therapy. The features were classified into positive and negative features based on the proportion of positive patches. There were 25 positive features and 13 negative features in total. Then we observed and analyzed the patches in each feature. The clustering and analysis process is shown in **Figure 2**.

### Generation of Key Feature

We evaluated patches of 38 features by 3 experts, and the characteristics of all features were showed in **Table 2**. Overall, these features were divided into two classes *via* image shape-based classification and tissue morphology-based classification. Consequently, 13 features were clustered by shape and 25 features were clustered by histology.

**Table 2:** 38 features and their patch numbers, histological morphology.

features	Ik	size	histological morphology
33	0.792793	1407	lymphocyte infiltration
15	0.652546	1348	lymphocyte infiltration and epithelium
9	0.626349	1542	epithelium and a little lymphocyte infiltration
35	0.588725	213	shape
10	0.57435	2209	muscle and connective tissue
23	0.570966	264	shape
19	0.568363	613	muscle and connective tissue
38	0.568304	694	muscle and connective tissue
26	0.565786	1605	muscle and light-colored connective tissue
1	0.563333	165	shape
18	0.55593	614	muscle and connective tissue
24	0.551183	369	shape
32	0.549619	169	shape
28	0.544116	706	shape
29	0.541248	1078	basal layer (excluding lymphocyte-rich areas)
16	0.53363	238	shape
36	0.528598	813	muscle and loose connective tissue
22	0.526346	1078	light-colored
37	0.524113	1783	muscle and loose connective tissue
5	0.517068	791	muscle and keratinized tissue
6	0.514626	165	keratinized layer
11	0.513952	2218	muscle and connective tissue
14	0.513298	1715	spinous and granular layers
8	0.507854	780	Muscle, adipose and connective tissue
25	0.503648	705	muscle and connective tissue
20	0.496371	719	muscle and connective tissue
3	0.491524	954	Muscle, adipose and connective tissue
12	0.464026	621	muscle and connective tissue
7	0.448971	156	shape
4	0.446255	1748	Muscle, adipose and connective tissue
34	0.430969	221	shape
13	0.429933	217	shape
21	0.416876	212	shape
17	0.364027	149	shape
31	0.361109	177	shape
2	0.299376	1938	areas containing blood vessels
27	0.241073	2144	loose connective tissue
30	0.237406	1289	epithelium and lamina propria (excluding lymphocyte-rich areas)

**Figure 3:**
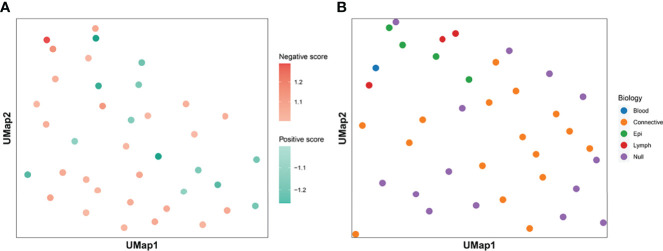
Clustering centroids. We showed the centroids of the 38 features. Panel **(A)** shows the effect of all features on the treatment effect. Darker red means that the feature has a greater significance of having a positive effect on immunosuppressive treatment. The darker the blue color, the greater the significance that the feature plays a negative role. Panel **(B)** shows the biological significance of all the features. The features dominated by muscle and connective tissue are distributed in the same area. Features which were clustered by shape were distributed in the periphery. The centers of epithelial and lymphocyte-dominated features were also close to each other.

We calculated the *I_k_* value of each feature. When *I_k_* is equal to 0.5, it means that the feature contains the same number of positive and negative patches, indicating no special effect on the treatment effect. If the value is greater than 0.5, the presence of the feature is considered as positive for treatment and is called positive feature; if the value is less than 0.5, the feature is considered as negative feature. We assigned all center points to different shades of color according to the value of *I_k_* (**Figure 3A**). The darker the color is, the more importance the feature means. **Figure 3B** shows the histological morphology of the features. The features of muscle and connective tissue were distributed in the same area. Features clustered by shape were distributed around. Epithelial and lymphocyte-dominated features’ centers were also close to each other.

**Figure 4:**
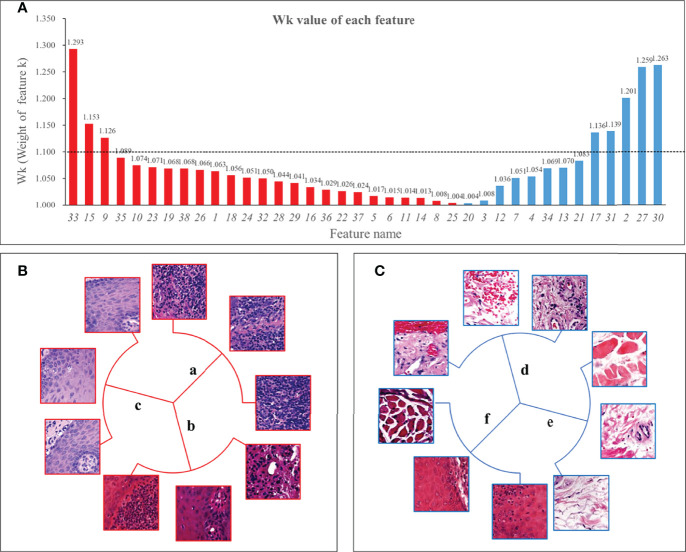
*W_k_* (Weight of feature k) of all features and 6 key features. Panel **(A)** showed the *W_k_* values of all features with the vertical axis taking the value of 1 to 1.35. The higher the value, the greater the impact on immunosuppressive treatment’s efficacy. We used 1.1 as the threshold and defined features with *W_k_* higher than 1.1 as key features. Panel **(B)** shows 3 positive features, 2 of which were mainly lymphocyte-infiltrated lamina propria and additionally contain a small amount of basal cell layer and spiny layer **(B.**a, **B**.b**)**. There was also a positive feature containing mainly the epithelial layer and, in addition, an infiltration of lymphocytes **(B**.c**)**. Among the negative features, one feature contained patches with the blood vessels **(C**.d**)**; one feature was loose connective tissue **(C.**e**)**; and one feature was the lamina propria, which contained part of the epithelial layer but not lymphocyte-rich areas **(C.**f**)**.

**Figure 4A** showed the weight *W_k_* of each feature. Among the 8 features exceeding 1.1, two features were classified by shape, so the remaining 6 features were defined as key features. In addition, we found that the features dominated by connective tissue and muscle tissue, whose *W_k_* were almost all less than 1.1, meant that the structure of muscle and connective tissue was of little significance for Immunosuppressive therapy.

### Histological Appearance of Key Features

Three of the six key features were positive and three were negative. The morphology of the features was shown in **Figure 4**. Two of the positive features (**Figure 4B**) were both mainly lymphocyte-infiltrated lamina propria, which also contained a small amount of basal cell layer and spiny layer (**Figures 4Ba, b**). The other positive feature containing mainly lymphocytes and epithelium. (**Figure 4Bc**). Of the negative features (**Figure 4C**), one feature mainly contained patches with the presence of blood vessels (**Figure 4Cd**); one feature was loose connective tissue (**Figure 4Ce**); and one feature was the lamina propria, which contained part of the epithelial layer but did not include lymphocyte-rich areas (**Figure 4Cf**).

### Key Features and Pathway Enrichment Analysis

**Figure 5:**
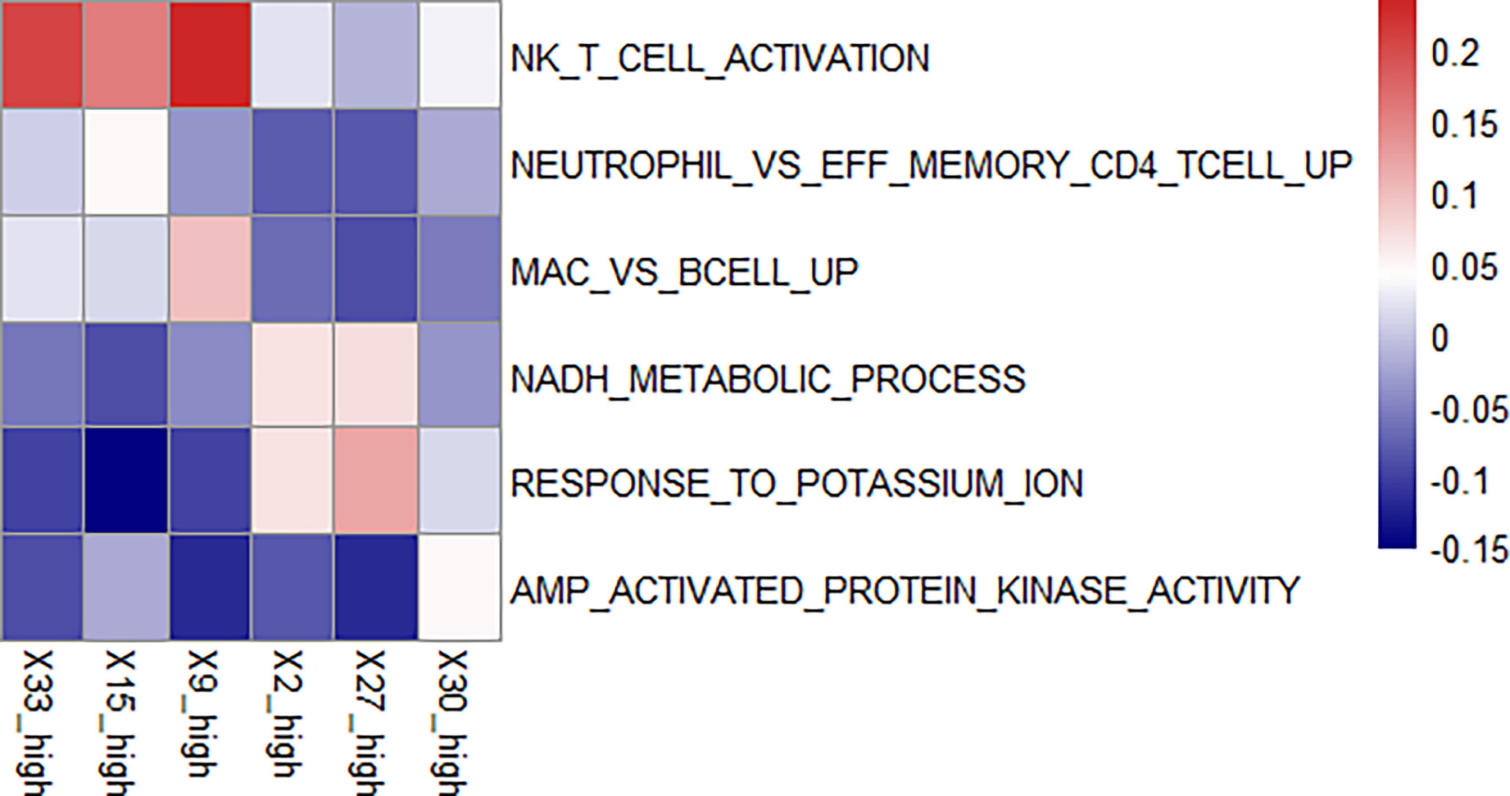
Enriched pathways in each feature. We found enriched pathways in the high expression group of each feature. Features 33, 15, and 9 all had lymphocyte-enriched pathways, but the types of lymphocyte were different, which may explain the lymphocyte infiltration group was divided into three features. Because of the enrichment of immune cells, immunosuppressive therapy has a good effect on the group with high expression of these three features. The biological phenotype of feature 2 was associated with the morphology of blood vessels, and the enrichment of NADH biochemical metabolic pathways was seen in the group with high expression. Feature 27 was associated with loose connective tissue, and its high expression group was enriched for the pathway of potassium ion overexpression. Feature 30 was enriched for kinase-related pathways in the high expression group. The presence of these three features has a suppressive effect on immunosuppressive treatment.

We used GSVA to determine the signaling pathways and functions, which were shown in **Figure 5**. Feature 30 was concentrated in the epithelium and lamina propria, and in the feature 30 high expression group, the kinase related pathway was enriched, and kinase expression was elevated. Kinases play important roles in current inflammatory and autoimmune diseases (34). Kinases can transduce signals from many cytokine receptors, inhibiting the effects of immunosuppressive treatments. Feature 27 contains the loose connective tissue. In the feature 27 high expression group, we found an enrichment in the potassium ion expression pathway. High levels of potassium maintain the “stem cell properties” of anti-cancer T cells, which have the ability to replicate themselves, but they cannot “grow” into killer immune cells (35). By keeping the T cells in this state, the tumor can avoid being attacked and continue to grow (36). Therefore, immunosuppressive therapy is ineffective when this pathway is highly expressed. In the characteristic 2 high expression group, the nicotinamide adenine dinucleotide (NADH) related pathway was enriched. The effect of immunosuppression may be reduced due to altered metabolism (37).

There was an enrichment of lymphocyte-related pathways in the three positive features. Feature 33 high expression group had enrichment of Natural killer T (NKT) cell, feature 15 high expression group had enrichment of neutrophil and T cell related pathway, and feature 9 high expression group had enrichment of B cell related pathway. The enrichment of immune cells resulted in increased efficacy of immunosuppressive treatment.

### Differential Pathway Enrichments of High and Low Expression Groups

**Figure 6:**
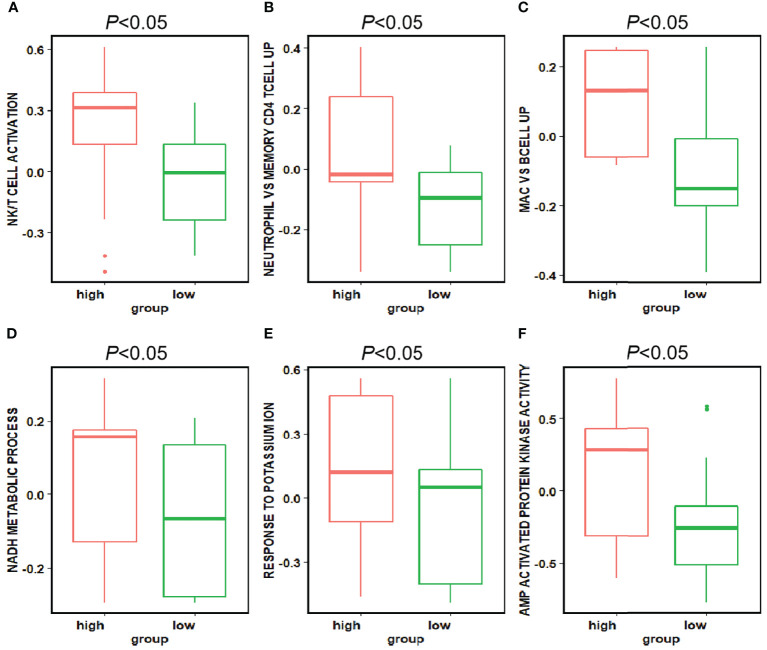
Differences in the pathways corresponding to the high and low expression groups of each feature. Panels **(A–F)** showed the differences in expression in the high and low expression groups of each feature in Figure 5 for the pathways enriched in the high and low expression groups of that feature, respectively. The *P-value* for the differences in pathway enrichment in the high and low expression groups for each feature is less than 0.05.

The difference of pathway enrichment was significant in high and low expression groups, for all key features (all *P* value less than 0.05). The result was shown in **Figure 6**. This implied that the key features we identified had a significant correlation between their histological manifestations and pathway enrichment.

### Predictive Models

We calculated the accuracy of the logistics regression models using the ten-fold validation method. The average model accuracy was 68. 53%, with a maximum accuracy of 92.31% and a minimum accuracy of 38.46%. the average AUC reached 0.722 (95% CI: 0.697-0.747).

We also compared the performance of SVMs. The average accuracy of the SVM model with 38 features was 76.68%, with the highest accuracy reaching 100% and the lowest 50%. the average AUC was 0.645 (95% CI: 0.637-0.652). the accuracy of the SVM was higher than the logistics regression overall, but the AUC value was lower because the results were categorical variables.

**Figure 7:**
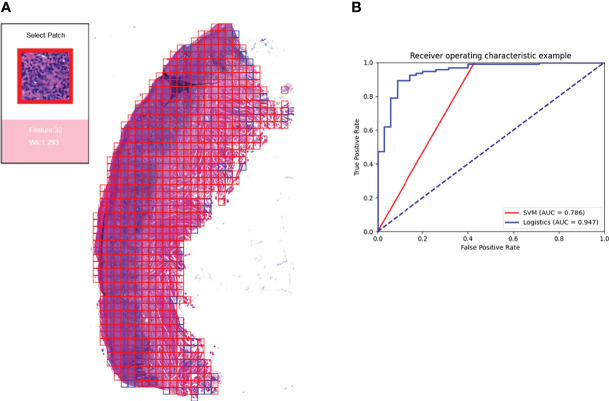
Prediction model and visualization results. We developed a model to predict the efficacy of immunosuppressive treatment based on a logistic regression model. Panel **(A)** shows the visualization of the prediction results. The patches belonging to positive features were added with red border, and those belonging to negative features are added with blue border. The box on the right shows the magnified morphology of the patch, the feature to which the patch belongs, and the *W_k_* value of the category. Panel **(B)** shows the ROC curves for the SVM model and the logistic regression model. Logistic regression has a higher AUC of 0.947.

For the ten-fold validation, we obtained different models. We tested the entire validation set using the best performing model, and the Logistic regression model achieved an accuracy of 90% with an AUC of 0.947. the SVM model had an accuracy of 88.46% with an AUC of 0.786. as shown in **Figure 7B**.

Based on the logistic regression model, we constructed a visual prediction system that could display the feature to which each patch belongs and the *W_k_* value of that feature, as shown in **Figure 7A**.

## Discussion

Our study implemented unsupervised learning from unlabeled pathological images, and we clustered and obtained features that can be interpreted. These features indicated the effect of OLP immunosuppression therapy. The size of the patch we used was sufficient for observed the content of patches. In addition to the features clustered by shape, each feature contained more than 500 patches, which makes it easy to summarize characteristics of the features. The key features contained histological features that were understandable to humans, which facilitates the selection of treatment regimens and the prediction of treatment effects. Features extracted by deep neural networks included not only human-identified findings, but also contain features that have not been noticed or discovered. Convolutional Neural Networks (CNNs) have been found to derive information from features that are undetectable by humans, such as determining patient age from X images of paranasal sinuses (38), predicting protein function from amino acid sequences (39).

The number of clusters was 38, which was exactly the number of WSIs as trained set. It could be a good solution for features extracted (25). A total of 38 histological features were obtained, these features can be classified into two main classes: one is classified by morphology. The patches of these features have common characteristics: they all contain blank areas, and the shape of the non-blank areas is uniform, rectangular, or triangular. The number of patches of these features is small, mostly less than 500, so they have little effect on the prediction results. The other is classified by histology. In this class, there were many features with muscle and connective tissue, and their *W_k_* were all in the range of 1 to 1.1, implying that such features had little effect on the treatment outcome. There were three key positive features, all of them contained lymphocyte-infiltrated lamina propria. Three negative features for treatment were blood vessels, loose connective tissue, and epithelial tissue separately. By analyzing the expression of relevant pathways, we confirmed the relationship between above features and their biological phenotype.

In this study, key features identified by deep neural network could be reasonable. We demonstrated a well-performing algorithm based on deep autoencoders (22) and with no need for manual labels information. In addition, our research has identified some of the features relevant to immunosuppressive therapy, and our findings were confirmed by pathway analysis. We also constructed a model to predict the effect of OLP immunosuppression treatment based on 38 features, and the logistics regression model achieved an accuracy of 90% with an AUC of 0.947. The model constructed based on our theory has a high accuracy for the prediction of treatment effect. However, the parameters we selected in the deep learning model were manually determined, which may not be the best choice. Changing the parameters or clustering methods may reveal more features. The prediction models we chose were the classic SVM and logistic regression. In future research, we will try to explore more potential models in prediction of diseases, such as neuralnet (40), and improve this tool to play a greater role in clinical diagnosis and treatment.

## Conclusion

OLP is a common disease of the oral mucosa. Immunosuppressive therapy is commonly used clinically, but patients have different response to treatment. There is a lack of in-depth clinical research on the reasons for the differences in efficacy. We obtained 38 features by training autoencoders to slice and data downscaling and clustering of pathology images. Through our analysis of biological phenotypes and pathways, we obtained 6 features that have a significant impact on efficacy. We also built a model to predict the efficacy of OLP immunosuppressive treatment based on these features. We did more than simply using WSI for classification and outcome prediction. More importantly, we acquired knowledge that can be interpreted and learned. This may be instructive for future research. Our study automatically obtains human interpretable features from pathological images, which could be an important reference for the clinical immunosuppressive treatment of OLP.

## Data Availability Statement

The datasets presented in this study can be found in online repositories. The names of the repository/repositories and accession number(s) can be found below: NCBI GEO, accession no: GSE204663.

## Ethics Statement

The studies involving human participants were reviewed and approved by Ethics Committee of West China Dental Hospital of Sichuan University. The patients/participants provided their written informed consent to participate in this study.

## Author Contributions

HX, QC, ZX, and QH contributed to conception and design of the study. QH and QC organized the database. ZX, DY, and JL performed the statistical analysis. ZX and QH wrote the first draft of the manuscript. QH, DY, QS, JL, and WL wrote sections of the manuscript. All authors contributed to manuscript revision, read, and approved the submitted version.

## Funding

This study was supported by grants from the National Natural Science Foundation of China (81730030) and Research funding for talents developing, West China Hospital of Stomatology, Sichuan University (RCDWJS2020-22).

## Conflict of Interest

The authors declare that the research was conducted in the absence of any commercial or financial relationships that could be construed as a potential conflict of interest.

## Publisher’s Note

All claims expressed in this article are solely those of the authors and do not necessarily represent those of their affiliated organizations, or those of the publisher, the editors and the reviewers. Any product that may be evaluated in this article, or claim that may be made by its manufacturer, is not guaranteed or endorsed by the publisher.
